# Critical contributions of pre-S1 shoulder and distal TRP box in DAG-activated TRPC6 channel by PIP_2_ regulation

**DOI:** 10.1038/s41598-022-14766-x

**Published:** 2022-06-24

**Authors:** Masayuki X. Mori, Ryo Okada, Reiko Sakaguchi, Hideharu Hase, Yuko Imai, Onur K. Polat, Satoru G. Itoh, Hisashi Okumura, Yasuo Mori, Yasushi Okamura, Ryuji Inoue

**Affiliations:** 1 Laboratory of Biomaterials and Chemistry, School of Medicine, University of Occupational and Environmental Health, 1-1, Iseigaoka, Yahatanishi-ku, Kitakyushu, Fukuoka 807-8555 Japan; 2 Human Information and Life Sciences, School of Health Sciences, University of Occupational and Environmental Health, Kitakyushu, Fukuoka Japan; 3 Laboratory of Molecular Biology, Department of Synthetic Chemistry and Biological Chemistry, Graduate School of Engineering, Kyoto University, Kyoto, Japan; 4 Department of Physiology, School of Medicine, Fukuoka University, Fukuoka, Japan; 5 Exploratory Research Center on Life and Living Systems/Institute for Molecular Science, National Institutes of Natural Sciences, Okazaki, Aichi Japan; 6 Department of Structural Molecular Science, SOKENDAI (The Graduate University for Advanced Studies), Okazaki, Aichi Japan; 7 Laboratory of Integrative Physiology, Department of Physiology, Graduate School of Medicine, Osaka University, Suita, Osaka Japan

**Keywords:** Biochemistry, Biophysics, Cell biology, Physiology, Structural biology

## Abstract

Phosphatidylinositol 4,5-bisphosphate (PI(4,5)P_2_ or PIP_2_) regulates the activities of numerous membrane proteins, including diacylglycerol(DAG)-activated TRPC3/6/7 channels. Although PIP_2_ binding is known to support DAG-activated TRP channel activity, its binding site remains unknown. We screened for PIP_2_ binding sites within TRPC6 channels through extensive mutagenesis. Using voltage-sensitive phosphatase (DrVSP), we found that Arg437 and Lys442, located in the channel’s pre-S1 domain/shoulder, are crucial for interaction with PIP_2_. To gain structural insights, we conducted computer protein–ligand docking simulations with the pre-S1 domain/shoulder of TRPC6 channels. Further, the functional significance of PIP_2_ binding to the pre-S1 shoulder was assessed for receptor-operated channel functions, cross-reactivity to DAG activation, and the kinetic model simulation. These results revealed that basic residues in the pre-S1 domain/shoulder play a central role in the regulation of PIP_2_-dependent gating. In addition, neutralizing mutation of K771 in the distal TRP box reversed the effect of PIP_2_ depletion from inhibiting to potentiating channel activity. A similar effect was seen in TRPV1 channels, which suggests that TRPC6 possesses a common but robust polarity switch mediating the PIP_2_-dependent effect. Overall, these mutagenesis studies reveal functional and structural insights for how basic residues and channel segments in TRP channels are controlled through phosphoinositides recognition.

## Introduction

Phosphatidylinositol 4,5-bisphosphate, also known simply as PI(4,5)P_2_ or PIP_2_, a phospholipid component of cell membranes, contributes to the activity of numerous molecules, including ion channels^1–3^. Among those are transient receptor potential (TRP) channels, which are known to sense diverse thermal, mechanical, and chemical stimuli and to be related to a variety of pathophysiological functions^4^. Nearly all TRP channels are positively or negatively regulated by PIP_2_^5–7^. For instance, reductions in membrane PIP_2_ levels inhibit most mammalian TRP canonical or classical (TRPC) channels^8–12^, while hydrolysis products of PIP_2_ such as DAG and polyunsaturated fatty acids (PUFAs) activate these channels^13,14^. Thus, the decrease in PIP_2_ level and resultant degradation products convey opposing signals to TRPC channels. These complicated but exquisite regulatory mechanism serves to control TRPC regulation in self-limiting and autonomic manner^8–10,12^. We previously showed that reducing PIP_2_ inhibits DAG-activated TRPC channels (TRPC3/6/7) channels, even in the presence of DAG, which indicates that the channel likely possesses distinct sites to interact with PIP_2_ and DAG, respectively^8,15^. However, no exact PIP_2_ binding/interaction site that could affect TRPC channel gating has yet been identified.

Our focus has been to gain knowledge into the molecular and the structural insight of the PIP_2_-interaction site on DAG-activated channels, especially TRPC6. TRPC6 plays an important role in the development of several fibrotic diseases including FSGS^16–18^, and does not conserve a classically established PIP_2_ binding domain, the Pleckstrin homology (PH)^19^. It has been reported that a complementary PH-like domain fragment in the N-terminal domain and the C-terminal domain serve as PIP_2_ binding sites in DAG-activated TRPC channels^20,21^. In addition, several other regions conserved in non-DAG-related TRP channels including the Ankyrin(ANK)-repeat domain (ARD) and TRP box, also reportedly bind PIP_2_^22,23^. Given this complexity, analysis that provides a broad perspective of PIP_2_ interaction sites is essential for a comprehensive understanding of the regulation of TRP channels.

In the present study, we investigated PIP_2_ interaction sites based on kinetic analysis upon the activation of DrVSP, a voltage-controllable PIP 5-phosphatase that is able to induce transient depletion of PIP_2_^24–26^. To do so, we applied this method along with extensive neutralizing mutation of the basic amino acid residues of TRPC6 channel. This enabled us to identify several residues critical for PIP_2_ binding that are broadly situated within the distal N-terminal region, ARD domain, S4-S5 linker, TRP box, distal TRP box, distal C-terminal regions, and pre-S1 domain/shoulder. Among these, the neutralization of basic residues in the pre-S1 domain/shoulder severely altered the deactivation and reactivation kinetics upon the PIP_2_ interaction. More specifically, channels mutated within the pre-S1 shoulder exhibited significant reductions in GPCR-activated TRPC6 current amplitudes. We also found that a mutation in the distal TRP box altered the effect of PIP_2_ depletion. Intriguingly, a basic residue mutation in the distal TRP box has been shown to switch PIP_2_ selectivity in the heat/capsaicin-activated TRPV1 channels^27^. These results imply that a common switching mechanism may underlay the actions of PIP_2_ recognition on TRP channels. Overall, our results provide valuable and fundamental insights into the mechanisms by which PIP_2_ regulates TRP channels and how PIPs and ion channels functionally interact with one another.

## Results

### Evaluation of PIP_2_ binding sites on the TRPC6 channel

**Figure 1:**
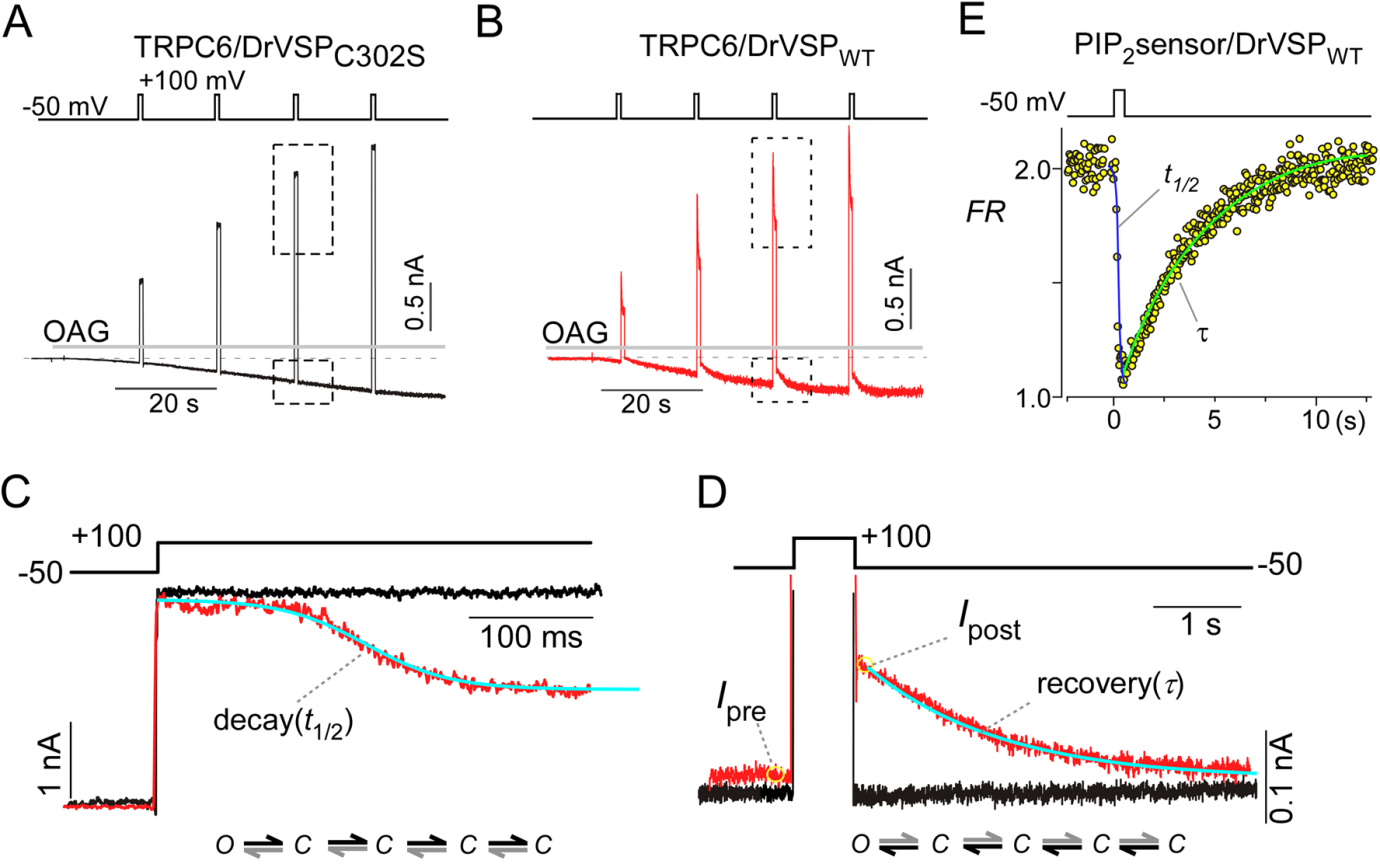
Kinetic analysis of PIP_2_ binding sites on TRPC6 channels. TRPC6 currents evoked by OAG (50 μM) were recorded in the whole-cell clamp mode from HEK293 cells co-transfected with enzyme-defective mutant DrVSP_C302S_ (**A**) or wild-type DrVSP (**B**). Strong depolarizations (+ 100 mV, 700 ms) were applied every 15 s (protocol displayed above). The areas enclosed by the dashed boxes are enlarged in (**C**,**D**). The deactivation (decay, *t*_1/2_) and reactivation (recovery, *τ*) kinetics were analyzed by fitting the equations described in the “Materials and methods” section (**C**,**D**, blue lines). Current inhibition upon PIP_2_ depletion was evaluated as the ratio of the currents after and before the depolarization (*I*_post_/*I*_pre_). Multistate gating diagrams are shown in the bottom. Channel deactivation and reactivation process by PIP_2_ involves four closed states, corresponding to each of the monomeric subunit in the tetrameric channel (**C**), with a single open state (O). (**E**) Kinetics of PIP_2_ depletion and replenishment upon the activation of DrVSP were measured with FRET.

**Figure 2:**
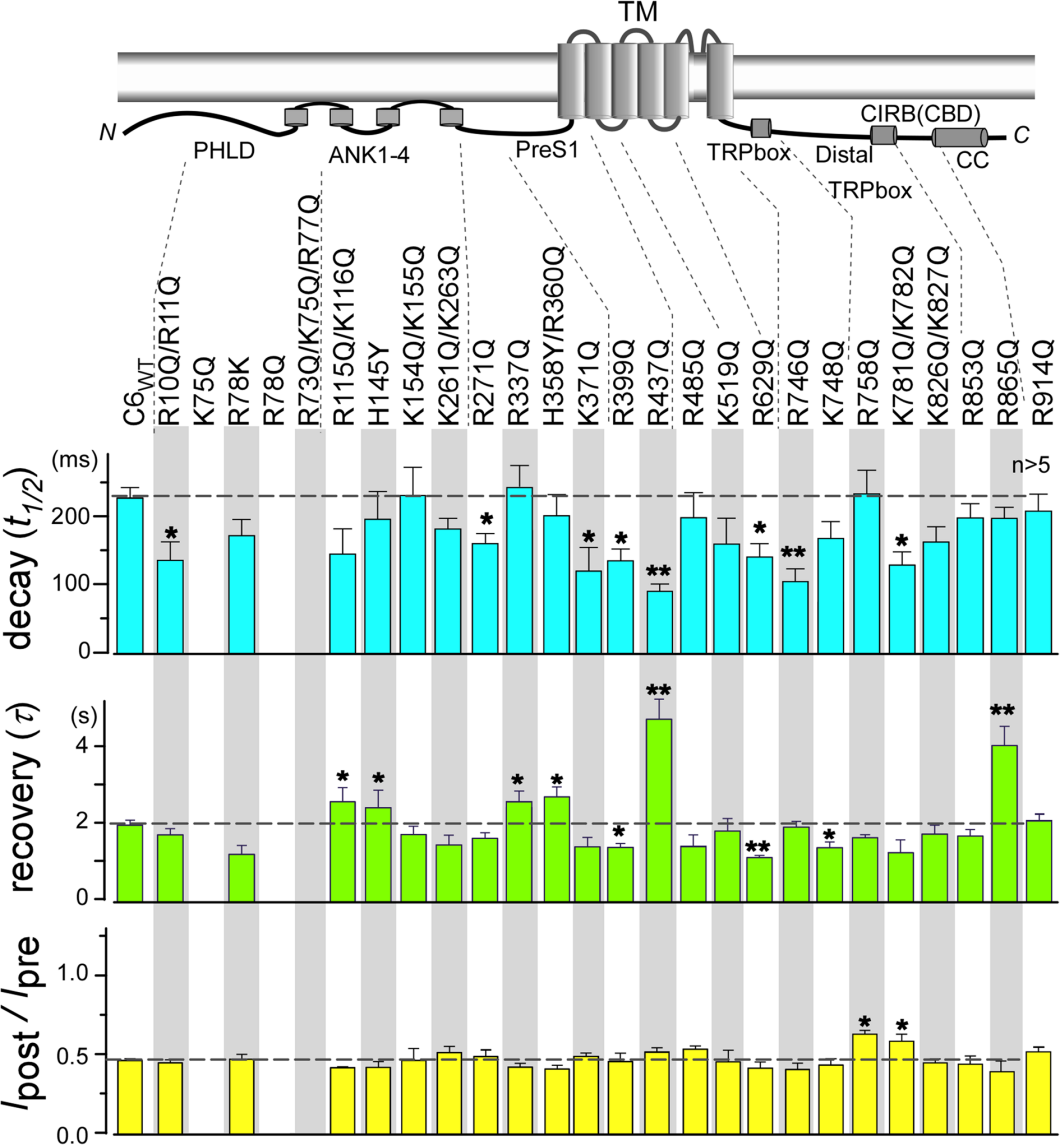
Screening for PIP_2_-associated residues through charge-neutralizing mutations in TRPC6 channels. Current decay (*t*_1/2_) and recovery (*τ*) (*n* = 5–10) and inhibition ratios (*I*_post_/*I*_pre_) are shown for each TRPC6 mutation. Asterisks indicate significant differences from TRPC6_WT_ values (*p < 0.05 or **p < 0.01).

To investigate the relationship between PIP_2_ binding and TRPC6 channel activity, we evaluated three features of OAG-induced TRPC6 currents in HEK293 cells. First one was the kinetics of TRPC6 channel deactivation (decay, *t*_1/2_) induced by DrVSP-mediated PIP_2_ depletion. DrVSP was activated by membrane depolarization to + 100 mV for 700 ms. Second was the kinetics of reactivation (recovery, *τ*) mediated by replenishment of PIP_2_ after repolarization to − 50 mV. These deactivation and reactivation kinetics can be used to estimate the dissociation and binding processes in reversible first order reactions. For wild-type TRPC6 (TRPC6_WT_, C6_WT_), decay (*t*_1/2_) was 229 ± 15 ms, while recovery (*τ*) was 2.01 ± 0.13 s (Figs. 1B–D and 2). The third feature examined was the functional impact of PIP_2_ dissociation (i.e., TRPC6 channel inhibition) elicited by DrVSP activation. This inhibition was expressed as the ratio of the current amplitudes before and after DrVSP activation (*I*_post_/*I*_pre_). This ratio was ~ 0.5 for TRPC6_WT_ (Figs. 1D and 2 bottom left). Co-expression of an inactive DrVSP mutant has no effect on TRPC6 currents (Fig. 1A,C,D). This indicates that TRPC6 channel activity led to no clear voltage-dependence, which was reported previously for other TRP channels^28–30^.

However, the kinetics of both the deactivation and the reactivation should reflect several distinct processes including the kinetics of VSP activation/inactivation, the time course of PIP_2_ dephosphorylation, and the altered channel gating upon PIP_2_ dissociation and bindings. Among those processes, we measured the dynamics of PIP_2_ alternation upon activation of DrVSP. The time courses of the depletion (*t*_1/2_) and replenishment (*τ*) of PIP_2_ were 135 ± 24 ms and 5.66 ± 0.48 s, respectively (Fig. 1E). This indicates a close correlation between PIP_2_ level and TRPC6 channel activity, and the kinetics of OAG-induced currents should reflect PIP_2_-dependent processes that affect TRPC6 channel functionality.

### Screening of PIP_2_ binding domains

TRPC6 channels possess nearly 90 positively charged (basic) residues in each subunit, among which we selected over 30 residues for the first screening based on following criteria: (1) residues were probably located on the intracellular side of the channel and (2) residues were clustered in the primary sequence with other positive residues. Mutant TRPC6 channels were co-expressed with DrVSP in HEK293 cells, and the membrane currents were measured by the whole-cell recordings. We found that following neutralization of basic residues in the distal N-terminal region (K75Q, R78Q, and R73Q/K75Q/R77Q), OAG no longer elicited any TRPC6-mediated currents (Fig. 2). This suggests that the distal N-terminal portion of the channel, where there is no available structure so far, may contribute to its translocation to the membrane, as suggested in the previous report^20^. The other 24 constructs tested carried OAG-induced currents, and 8 out of them exhibited a significantly faster decay upon PIP_2_ depletion than TRPC6_WT_ (Fig. 2, upper, blue bars). Among those, R437Q and R746Q mutants were the fastest (92 ± 10 ms (*n* = 6) and 118 ± 13 ms (*n* = 9), respectively).

On the other hand, the recovery of TRPC6 current from DrVSP-mediated inhibition, with restoration of PIP_2_ was significantly accelerated or delayed in 9 out of 26 constructs (Fig. 2, middle, green bars). The current recovery was markedly delayed in R437Q and R865Q mutants, 4.78 ± 0.53 s and 3.70 ± 0.51 s, respectively. These mutations are respectively located in the pre-S1 domain/shoulder and calmodulin/inositol-1,4,5-trisphosphate receptor binding domain (CIRB)^31^, the latter is also known as PIP_2_/calmodulin binding domain^21,32^. Only the single mutation R758Q and double mutation K781Q/K782Q reduced the effect of PIP_2_ depletion, as indicated by the weak inhibition ratio (*I*_post_/*I*_pre_), (Fig. 2, bottom, yellow bars). However, none of the mutations led to more than 50% current inhibition upon PIP_2_ depletion. This suggests that there is a 50% PIP_2_-independent component in the OAG-induced currents or the maximal efficacy of DrVSP to deplete endogenous PIP_2_ may not be 100%, possibly due to fast replenishment of PIP_2_ in some compartmentalized area.

### Pre-S1 is a PIP_2_ binding domain

**Figure 3:**
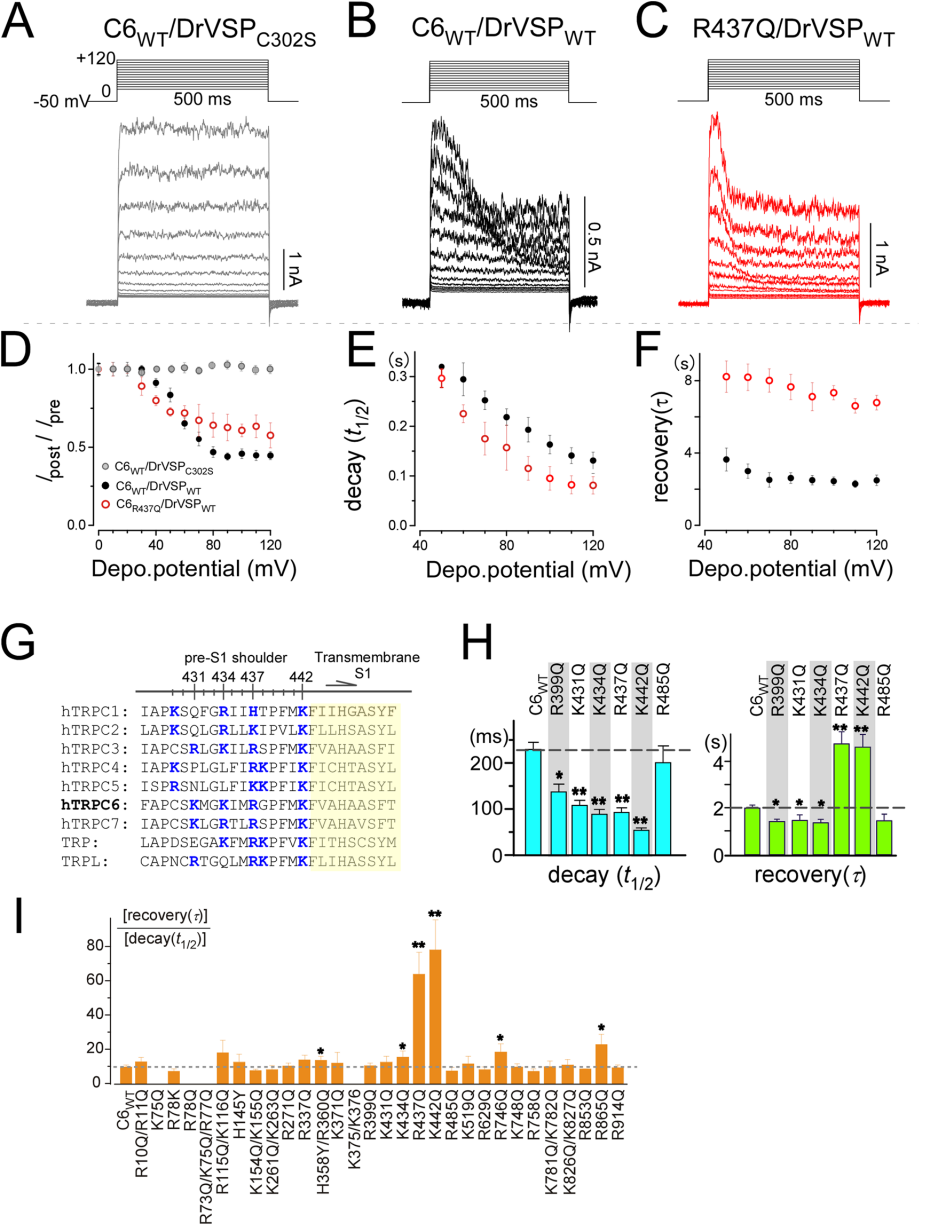
Basic residues in the pre-S1 domain/shoulder are critical for PIP_2_ binding. Voltage-step pulses in HEK293 cells co-expressing TRPC6_WT_ and DrVSP mutant (**A**) or TRPC6_WT_ or TRPC6_R437Q_ and DrVSP_WT_ (**B**,**C**). (**D**–**F**) Summary of the voltage-step pulse results. (**D**) *I*_post_/*I*_pre_, (**E**) PIP_2_ deactivation kinetics, (**F**) PIP_2_ reactivation kinetics. (**G**) Sequence alignment of the pre-S1 shoulder from human TRPC and *Drosophila* TRP channels. Positively charged residues are shown in blue. The numbering is based on human TRPC6 channel. (**H**) PIP_2_ deactivation and reactivation kinetics after charge neutralization mutation of the TRPC6 pre-S1 shoulder. The voltage pulse protocol is identical that in Fig. 2. (**I**) Ratio of the reactivation/deactivation kinetics for estimation of dissociation strength. The R437Q and K442Q mutants showed significantly reduced interaction with PIP_2_ (*p < 0.05 or **p < 0.01).

In contrast to the aforementioned constructs, R629Q (in S4–S5 linker) and K748Q (within TRP box) mutants exhibited accelerated recovery from the inhibition (1.17 ± 0.05 s and 1.47 ± 0.11 s, respectively), suggesting that these basic residues may have less contribution to rebinding of PIP_2_. Thus, among the first screened mutants, the pre-S1 domain/shoulder mutation R437Q exerted the most critical effect on both the decay and the recovery of TRPC6 channel currents (Fig. 2). This finding was further confirmed using the depolarizing step-pulse protocols over a wide range of membrane potentials (Fig. 3A–F). The results revealed that throughout the membrane potential tested, the R437Q mutation decays faster and recovers more slowly than TRPC6_WT_.

**Figure 4:**
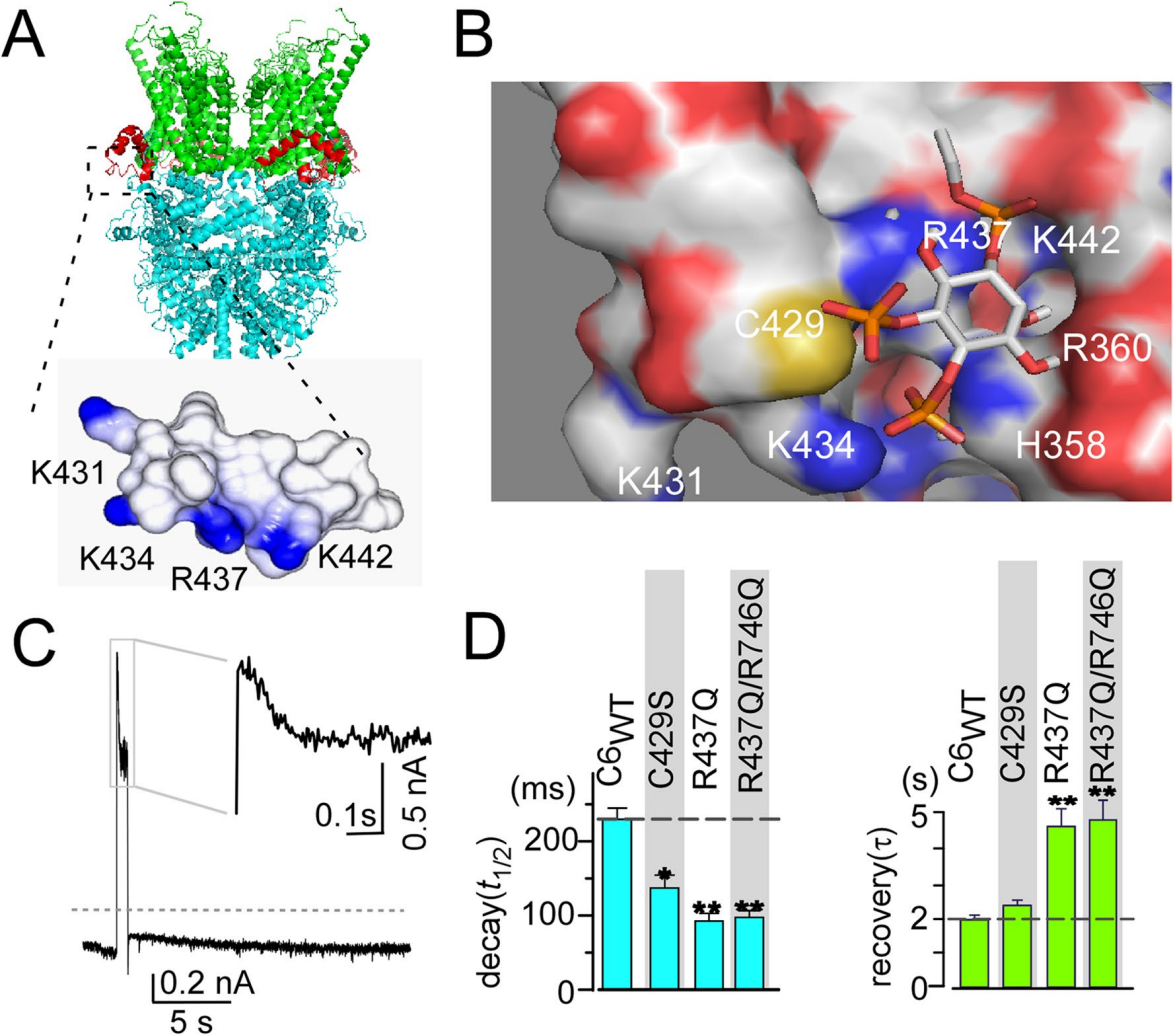
Simulation of PIP_2_ docking to the pre-S1 domain. (**A**, top) Ribbon diagram of TRPC6 (PDB: 6UZ8^33^). The pre-S1 domain is depicted in red. (Bottom) Surface plot of the pre-S1 shoulder. (**B**) Surface representations of the arrangement of the short PIP_2_ chain and the pre-S1 domain/shoulder with the linker segment between the ARD and pre-S1. (**C**) Trace showing the current through the double mutants R437Q/R746Q channel upon the activation of VSP. Gray dashed line indicates zero current level. (**D**) Summary of C429S and R437Q/R746Q kinetics to VSP activation for comparison with TRPC6_WT_ and R437Q (*n* = 5 and *n* = 4 for C429S and R437Q/R746Q, respectively). *p < 0.05 or **p < 0.01.

Because the pre-S1 shoulder is enriched in basic residues (Fig. 3G), this prompted us to conduct a second mutational screening focused on the pre-S1 shoulder, which revealed the importance of K442, in addition to K431 and K434. The time constants for the decay and recovery of K442Q were 54 ± 11 ms and 3.71 ± 0.53 s, respectively (Fig. 3H). Amino acid sequence alignment of the pre-S1 domain/shoulder of mammalian TRPC channels shows identical positively charged residues at sites equivalent to R437 and K442 (Fig. 3G). This indicates that R437 and K442 in the pre-S1 shoulder play an essential role in PIP_2_ binding to TRPC channels, including TRPC6. Moreover, the contributions of basic residues within the TRP box were also undoubtful in the kinetic analysis, which showed R746Q to produce the fastest current decay among the tested mutations (Fig. 2). To understand whether the pre-S1 shoulder and the TRP box exert a cooperative effect on PIP_2_ binding, we examined the effect of R437Q/R746Q double mutations. The decay and recovery kinetics of this double mutant were indistinguishable to R437Q mutant (Fig. 4C,D), indicating that the contribution of R437 is dominant to R746 for PIP_2_ binding, rather than showing an additive effect.

To compare PIP_2_-affinity to respective mutants, we calculated the ratio of the recovery (τ) and decay (*t*_1/2_) as a value related to the dissociation constant (Fig. 3I). Based on this estimation, the R437Q and K442Q channels exhibited nearly five to eight-fold less affinity for PIP_2_ than the TRPC6_WT_ channel (Fig. 3I). Given that the dissociation constant for PIP_2_ binding to TRPC6_WT_ was previously reported to be 2 µM^15^, we propose that those for PIP_2_ binding to the pre-S1 domain/shoulder mutants (R437Q and K442Q) are greater than 10 µM.

### PIP_2_ binding to the pre-S1 domain/shoulder, linker domain, and TRP box

Recent cryo-EM structures of TRPC3/6 channels showed that the pre-S1 domain is exposed to the outside of the channel complex^33,34^. Within the pre-S1 domain, positively charged residues are situated every three or five amino acids (K431, K434, R437, K442) to form an amphipathic helix termed the “pre-S1 shoulder” (Fig. 4A). Intriguingly, the pre-S1 shoulder is positioned at the inner surface of the membrane, where it encounters the residues H358 and R360. Neutralizing mutations of those residues slows the recovery, implying weaker PIP_2_ affinities for TRPC6 channel (Fig. 2). This suggests the basic residues in the pre-S1 shoulder as well as the H358/R360 residues are crucial for recognition of PIP_2_.

To confirm this idea, we performed a docking simulation with PIP_2_ and the pre-S1 shoulder using the cryo-EM structure of TRPC6 (PDB 6UZ8). Within the simulation, the inositol head of PIP_2_ was positioned at a surface pocket surrounded by the pre-S1 shoulder and H358/R360 residues. The TRPC6_WT_ model exhibited the highest docking energy (-6.0 kcal/mol), while R437Q and K442Q pre-S1 shoulder mutants exhibited somewhat lower docking energies (− 5.4 and − 5.6 kcal/mol, respectively). Intriguingly, the docking free energy between PIP_2_ and H358/R360 mutant was clearly reduced (− 4.9 kcal/mol). These results reflect the decreased affinity between PIP_2_ and the mutant channels. Indeed, we also tested a point mutation at a cysteine residue (C429), because it is located in the pre-S1 domain and is near the center of PIP_2_-docking area. Although the docking energy of the C429S mutant was no lower than that of the wild-type channel (− 6.0 kcal/mol), the actual decay was significantly accelerated (121 ± 17 ms, Fig. 4D). These observations further confirm that the pre-S1 shoulder and H358/R360 residues contribute critically to PIP_2_ binding.

### Functional role of the pre-S1 domain/shoulder

**Figure 5:**
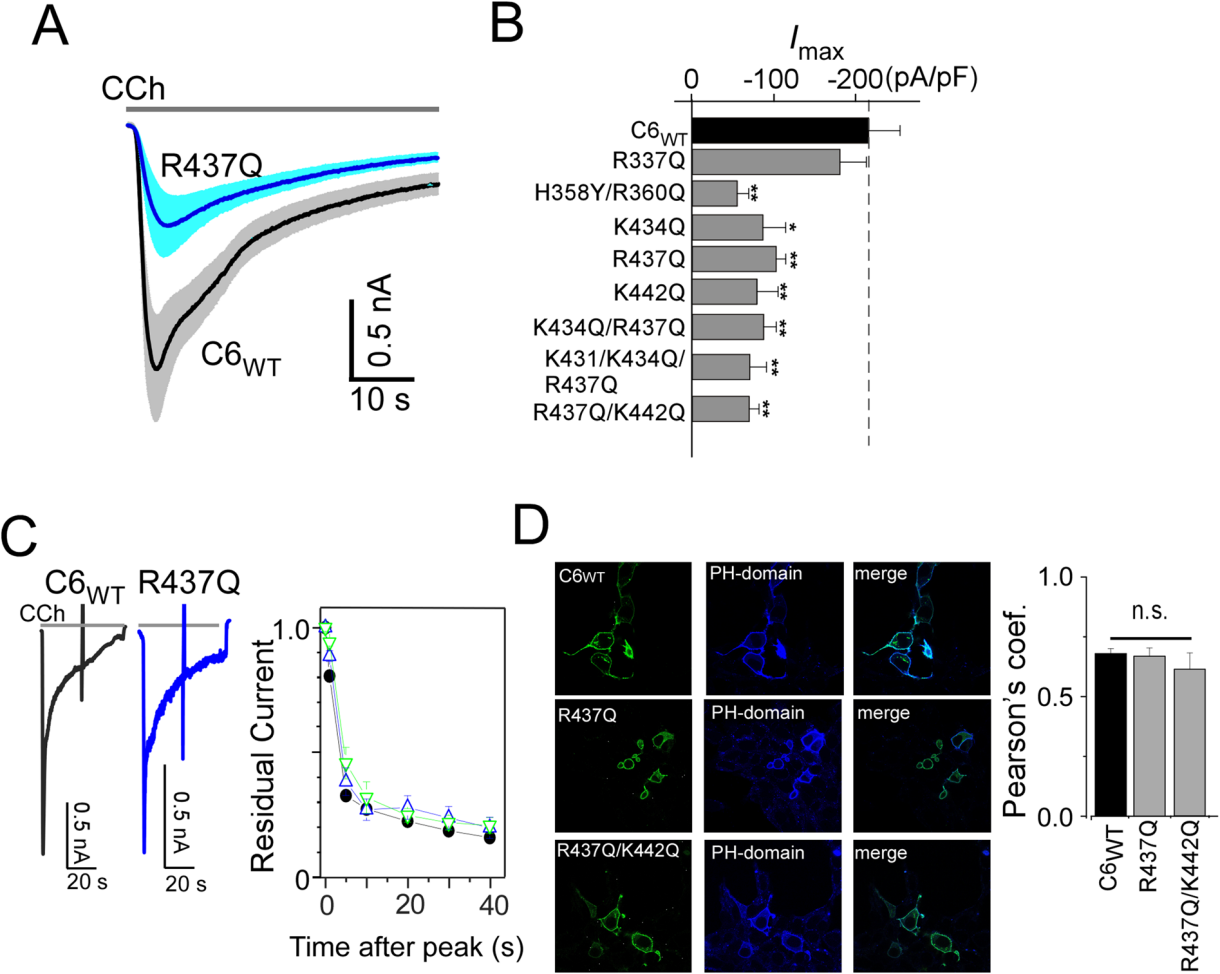
Functional role of the pre-S1 domain/shoulder. (**A**) Averaged traces of TRPC6 currents induced by CCh (100 μM). Black: TRPC6_WT_, blue: R437Q mutant. Light gray and blue regions indicate SEM. (**B**) Summary of receptor-operated current densities obtained with various TRPC6 mutants (*n* > 5). (**C**) Ca^2+^-dependent inactivation. Representative traces for the CCh response in TRPC6_WT_ (black) and R437Q (blue) under the low Ca^2+^ buffering condition (1 mM EGTA in the intracellular solution). R437Q trace was scaled to match WT traces (at scale with bar). Residual currents after the peak show identical inactivation profiles with the TRPC6_WT_ (right panel, black circles), the pre-S1 mutants (R437Q (*n* = 7), and R437/K442Q (*n* = 5) for blue and green triangles, respectively. (**D**) Confocal microscopic analysis of the distribution of pre-S1 mutants. Left: CFP-fused channel. Middle: YFP-fused PH domain. Right: Merged images. Pearson’s correlation coefficient (*n* > 10). *p < 0.05 or **p < 0.01.

For evaluation the functionality of the PIP_2_ binding pocket identified in the docking simulation, TRPC6 channels were co-expressed with muscarinic receptors, which were then stimulated with a muscarinic receptor agonist carbachol (CCh). We found that the maximum current density upon receptor stimulation was significantly suppressed by single or double mutations, H358Y/R360Q, R339Q, K434Q, R437Q, and K442Q (Fig. 5A,B). Moreover, the extents of suppression elicited by these mutations as well as those by different combinations of double or triple mutations K434Q/R437Q, R437Q/K442Q, and K431Q/K434Q/R437Q were nearly identical. This confirms that the surface pocket for the PIP_2_ interaction is crucial for receptor-activated channel activity. To then assess the importance of the pre-S1 domain/shoulder for channel localization in the cell membrane, we used confocal microscopy to compare the localizations of TRPC6_WT_ and R437Q and R437Q/K442Q mutants fused with cyan fluorescence protein (CFP) based on their co-localization with the yellow fluorescence protein (YFP)-fused PH-domain sensor. Co-localization of the channel and PH-domain after transfection into HEK293 cells did not statistically differ between the wild-type and mutant TRPC6 channels (Pearson’s correlation coefficient = 0.68 ± 0.02 for WT and 0.61 ± 0.07 for R437QK442Q, Fig. 5D). This suggests the pre-S1 domain/shoulder mainly contributes to channel functionality, but not to PIP_2_-dependent membrane localization.

PIP_2_ binding to TRPC channels allows the channels availability, and the less binding is possible to induce channel dysfunctionality, vice versa. It has been shown that impairment of Ca^2+^-dependent inactivation of TRPC6 channels is a cause of FSGS^32^. We therefore measured the receptor-activated TRPC6 current inactivation of the pre-S1 mutants under low Ca^2+^ buffering conditions. As shown in Fig. 5C, R437Q, R437/K442Q mutants and TRPC6_WT_ exhibited almost identical residual currents. Thus, binding of PIP_2_ to the pre-S1 domain/shoulder is not directly involved in Ca^2+^-dependent inactivation and may not account for the pathogenesis of FSGS.

**Figure 6:**
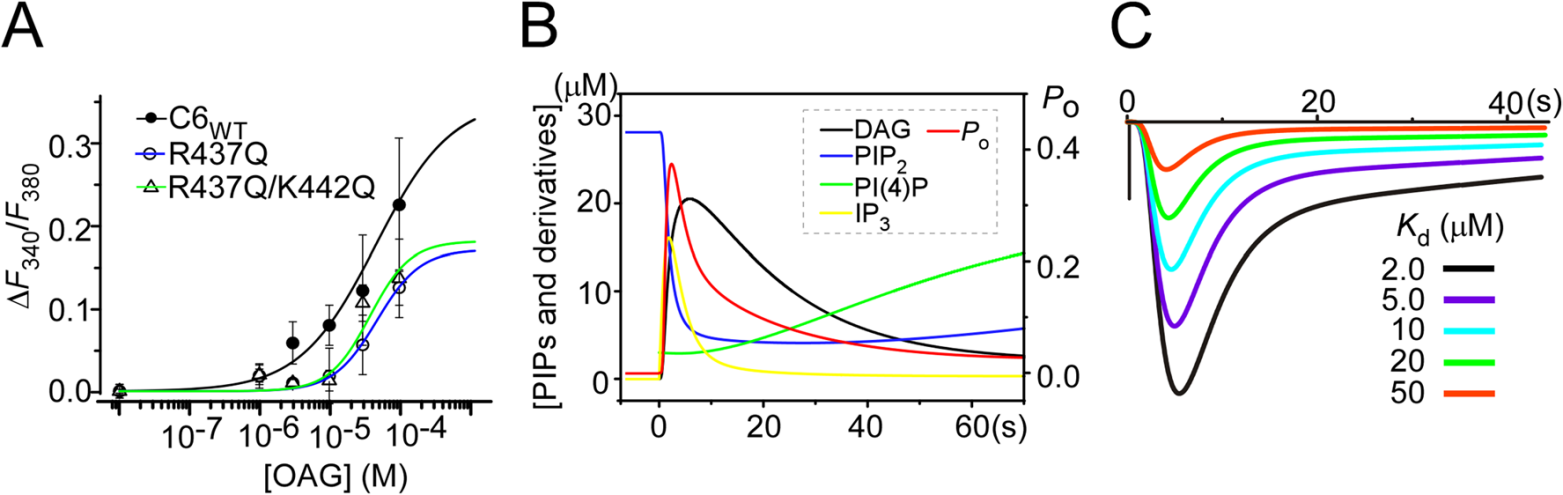
Dose–response to DAG and kinetic simulation. (**A**) Dose–response relationship for OAG in TRPC6-expressing cells obtained by Ba^2+^ influx imaging using Fura-2 (*n* = 12–25 for each data point). (**B**) The simulated time course of concentration of PIPs signaling products and open probability (*P*o) of TRPC6 channels. (**C**) Current traces generated in a PIP_2_-DAG model simulation in which the dissociation constants for PIP_2_ binding to TRPC6 channels were varied. Wild-type TRPC6 has ~ 2 µM of dissociation constants for PIP_2_ binding^15^. The black scale bar indicates 0.5 nA.

In addition, here and previously, we demonstrated that reducing PIP_2_ inhibits channel activity, even in the presence of DAG^8^. We therefore tested how PIP_2_ affects the potency of DAG to activate TRPC6 channel by comparing the concentration-dependent effects of OAG on TRPC6_WT_ and pre-S1 mutants. Figure 6A shows the concentration dependence of the responses of R437Q, R437Q/K442Q mutant and TRPC6_WT_ channels to OAG. This dependency was investigated by ratiometric Fura-2 photometry^35^. The normalized dose–response data were best fit to the Hill equations with EC_50_ values of 48, 37 and 46 μm and the coefficients (*n*) of 1.4, 1.5 and 0.8 for R437Q, R437Q/K442Q, and TRPC6_WT_, respectively. Thus, mutations in the pre-S1 domain/shoulder cause practically no change in EC_50_ values. This suggests that PIP_2_ binding does not significantly affect DAG binding but instead may affect allosteric activation by DAG.

We used our previously described simulation model, to evaluate how PIP_2_ binding affects receptor-activated TRPC6 channels^15^ (Fig. 6B). By reducing the channel’s binding affinity for PIP_2_ while keeping its affinity to DAG, the amplitude of a simulated current was gradually decreased (Fig. 6C). When PIP_2_-channel affinity was reduced by one-fifth, the current amplitude was reduced by half (Fig. 6C, black vs. blue traces). This result is consistent with those obtained with the R437Q and K442Q mutants, which exhibited reduced PIP_2_ affinities and current densities (Figs. 3I and 5A,B). Taken together, these findings suggest that reducing PIP_2_ binding affinity decreased TRPC6 channel activity without altering its membrane localization. Our study therefore uncovers that the pre-S1 domain/shoulder of TRPC6 makes the critical contribution to PIP_2_ binding.

### K771 mutation in the distal TRP box alters the effect of PIP_2_

**Figure 7:**
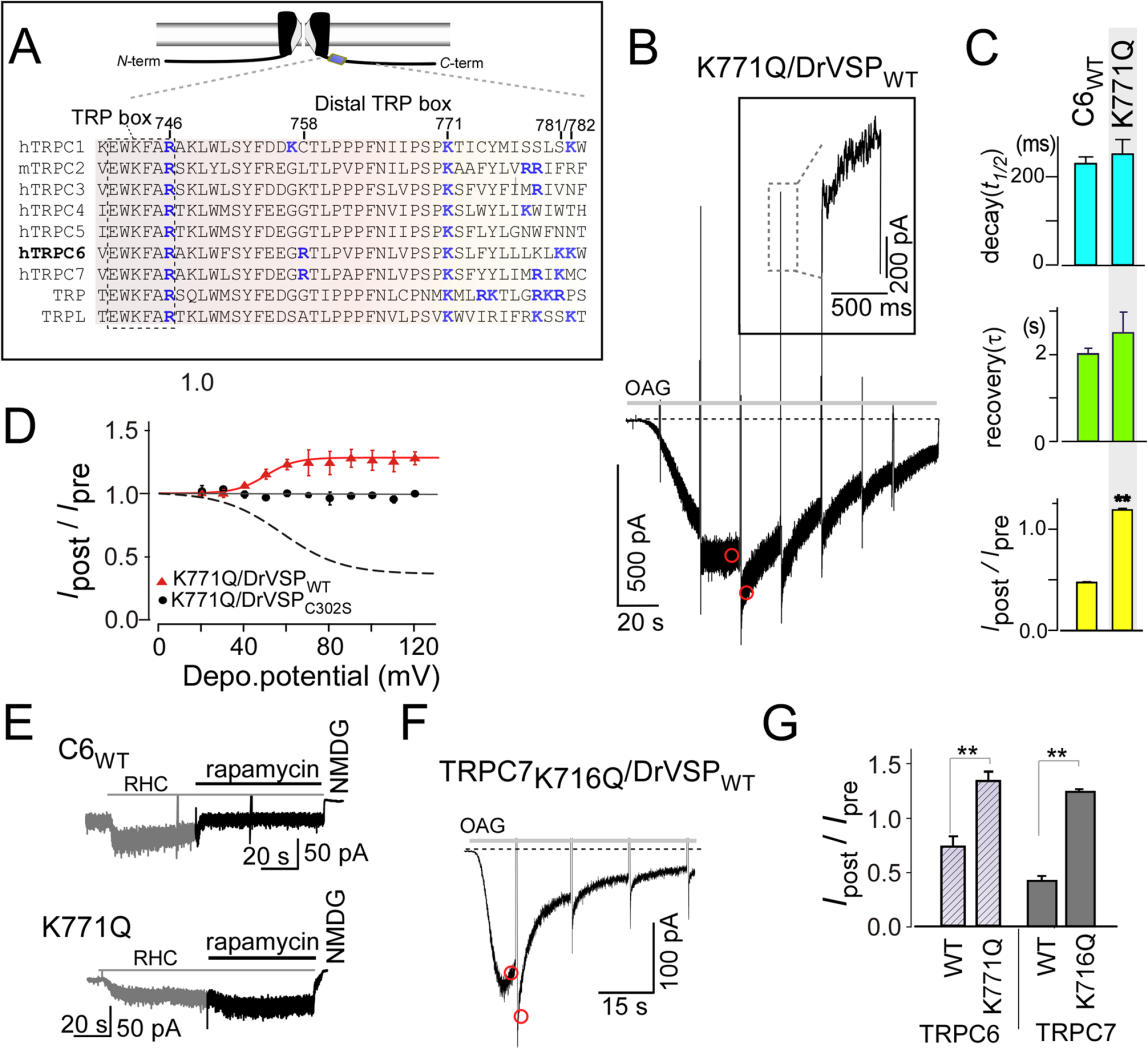
K771Q mutation reversed the effect of PIP_2_ depletion. (**A**) Sequence alignment of TRP box to distal TRP box in human TRPC/drosophila TRP channels. Numbering corresponds to hTRPC6 amino acid sequence. Critical basic residues for PIP_2_ regulation are indicated in blue bold. (**B**) 50 μM OAG-induced currents recorded from HEK297 cells expressing K771Q and DrVSP_WT_. The inset shows an enlargement of the outward current. (**C**) Summary of deactivation (top), reactivation (middle) kinetics, and *I*_post_/*I*_pre_ (bottom) (for K771Q *n* = 5). (**D**) Result of the voltage-step protocol with K771Q and DrVSP_WT_ showed in red circles. Depolarizing pulses were stepped from 0 to + 120 mV in 10-mV increments. Black circles depict the ratio of *I*_post_/*I*_pre_ from VSP mutant DrVSP_C302S_ with K771Q. These results indicate no visible voltage-dependent activation and PIPs selectivity alternation effect which is reported in Ci-VSPs^38,39^. The dashed line shows response with TRPC6_WT_/DrVSP_WT_ (data is identical to Fig. 3D). (**E**) Response to rapamycin (10 μM)-induced PIP_2_ reduction in cells expressing TRPC6_WT_ (top) and K771Q (bottom). Currents were evoked by RHC-80267 (100 μM) (F) TRPC7 K721, which is the equivalent amino acid to K771 in TRPC6, mutation (K721Q) potentiated the channel opening upon DrVSP activation. OAG (50 μM) was applied to induce the currents. (**G**) Summary of the polarity switch induced by rapamycin (diagonal line bars, *n* = 4, respectively) on TRPC6 channels and by effect of DrVSP_WT_ on TRPC7_K721Q_ (gray bars, *n* > 4). **p < 0.01.

As shown in Fig. 2, upon the PIP_2_ depletion, R758Q and K781Q/K782Q mutations significantly decrease the inhibition ratio (*I*_post_/*I*_pre_) as compared to TRPC6_WT_. These residues are located within the distal TRP box, the importance to the thermal sensitivity of which site was shown in earlier studies of PIP_2_-mediated regulation of TRPV1 channels^36,37^, but lacking such information for TRPC channels. We therefore questioned whether the other basic residues located between R758 and K781/K782 might affect the effects of PIP_2_ depletion. Unexpectedly, when K771, an evolutionary highly conserved residue, was mutated to glutamine, the resultant OAG-induced currents were potentiated after activation of DrVSP (*I*_post_/*I*_pre_ = 1.24 ± 0.13 *n* = 7, Fig. 7A–C). This potentiation was observed repeatedly during the recording period (Fig. 7B). By contrast, no potentiation of K771Q-mediated currents was seen when the inactive DrVSP was co-expressed (Fig. 7D, black circles). This eliminates the possibility of a gain-of-function effect on the voltage-dependent activation.

Moreover, using the *rapamycin*-inducible FKBP12-Inp54p system^40,41^, which also depletes PIP_2_ by the membrane recruiting of specific inositol 5-phosphatase, the potentiation of TRPC6 currents still occurred (Fig. 7E,G). To explore if this reversed effect of PIP_2_ depletion can be generalized to other TRPC channels, we tested the effect of neutralization on the equivalent residue (K716) in TRPC7. As shown in Fig. 7F, the TRPC7 K716Q mutant exhibited a potentiated channel activity upon PIP_2_ depletion. The potentiation ratio for TRPC7_K716Q_ was 1.28 ± 0.06, which is nearly identical to that of TRPC6_K771Q_. These results suggest that certain basic residues in the distal C-terminal region (TRP box) act to control the polarity of PIP_2_-dependent effects on TRPC6/7.

## Discussion

PIP_2_ is known to regulate most TRP channels^42^. Earlier studies showed that reductions of PIP_2_, either through dephosphorylation by VSP or hydrolysis by PLC, suppress TRPC channel activity^8,10,12,15,42^. PIP_2_ has three phosphate groups, and their negative charges likely form an interactive network through electrostatic interactions or salt bridges with basic residues in target molecules^43^. Up to now, however, no PIP_2_ interaction site for TRPC channel’s activity had yet been determined.

TRPC6 contains over 400 basic residues in the tetrameric complex. In the present study, we introduced neutralizing mutations to various selected basic residues. As a result, 11 of the 29 mutated channels exhibited accelerated deactivation (decay, *t*_1/2_) upon VSP activation, while 12 of the 29 channels showed altered reactivation (recovery, *τ*). In total, 17 mutated channels exhibited altered deactivation or reactivation kinetics, though only the R437Q and K442Q mutants showed significant effects on both deactivation and reactivation to reduce the effective PIP_2_ binding affinity. In previous reports, we have determined decay and recovery constants of TRPC6 and TRPC7 channels^8^. Based on this data, the ratio of recovery (τ)/decay (*t*_1/2_) showed good agreement with the rank order of *K*_d_ values for PIP_2_-TRPC6 and PIP_2_-TRPC7 channels, which are 2 and 5 μM, respectively^15^. In current report, the kinetic ratio of TRPC6_WT_ was 10.01, and R437Q and K442Q were 47.76 and 77.13, respectively (Fig. 3I). Thus, dissociation constants of R437 and K442 to PIP_2_ could be decreased to 5–8 folds. However, this estimation can only be effective under a limited condition, where the channel gating kinetics is equal among the respective mutants. Therefore, although it is difficult to obtain the exact dissociation constant, we could still compare relative rank order for PIP_2_ affinity, and emphasize the importance of pre-S1 domain for PIP_2_ binding. This pre-S1 domain in TRP channels has received particular attention because of the abundance of electrophilic amino acids and its structural proximity to the TRP box^44–49^. The pre-S1 domain contains the pre-S1 elbow and pre-S1 shoulder, the latter of which forms an amphipathic short helix along the inner leaflet of the cell membrane (Fig. 4A). Within this helix, side chains of basic residues, including K431, K434, R437 and K442, extend into the cytoplasm, making them suitable for PIP_2_ binding. The docking simulation also supports the importance of basic residues in the pre-S1 shoulder. Nevertheless, an additional pre-S1 shoulder mutation in cysteine at 429 to serine (C429S) did not cause any difference in the docking score as expected, but the VSP-based experimental results indicated the acceleration of PIP_2_ dissociation kinetics (Fig. 4C). These inconsistent observations may be due to more direct exposure of the pre-S1 domain to the solvent caused by Cys to Ser mutation, which should greatly affect the recognition of PIP_2_^50^.

We also observed that recovery kinetics was slowed in the H358Y/R360Q mutant (Fig. 2). Both H358 and R360 are located within the linkage domain between the ARD and the pre-S1, and are facing basic residues in the pre-S1 shoulder (Fig. 4B). These residues likely facilitate the interaction between PIP_2_ and the pre-S1 shoulder, as supported by the result of docking simulation. In the receptor activation experiments, the current density was lower with the H358Y/R360Q mutant than TRPC6_WT_ (Fig. 5B).This highlights the idea that the positively charged H358 and R360 cooperatively contribute to PIP_2_ binding with pre-S1 domain/shoulder. This may be consistent with a recent structural study showing that in TRPM8 channels, the head group of PIP_2_ is situated within a pocket coordinated by the pre-S1, TRP box, the linker region between the pre-S1 and the TRPM homology region (MHR) domain^49^. This TRPM8-PIP_2_ binding may be similar to TRPP channels where pre-S1 and TRP box-like domain coupling supports the negative effect of PIP_2_^48^. However, in TRPM8 and TRPV5, the S4-S5 segment also forms part of the PIP_2_ binding site^47,49^. This does not seem to be the case in TRPC6, as the mutation (R629Q) in this segment had faster inhibition, but also showed faster recovery from DrVSP inhibition (Fig. 2). This residue, and the other untested positive charge in this region seems to be not close enough to the pre-S1 PIP_2_ interacting residues. Consequently, the contribution of the pre-S1 domain/shoulder may be greater than in TRPM8 and TRPV5. Thus, we assume that in TRPML and PI(3,5)P_2_ structures, the location of the PIP_2_ near pre-S1 (though missing in the structure) is close to TRPC6 channel^47^. To summarize this mutational study in the structural aspect, we have mapped residues either only for decay (*t*_1/2_) or recovery (τ) and both are altered in the TRPC6 channel structure (Supplemental Fig. 1). This mapping reveals that basic residues locate near the inner leaflet contribute to decay (*t*_1/2_). Contrary to these, residues contributing for recovery (τ) locate in the intracellular domain. These observations indicate that basic residues near the transmembrane would retain PIP_2_ binding, and those in the intracellular region of the channel would attract PIP_2._

To investigate the pathophysiological importance of PIP_2_ binding to the pre-S1 domain/shoulder, we assessed its contribution to Ca^2+^-dependent inactivation of the TRPC channel. It has been argued that the dissociation of PIP_2_ could be involved in the desensitization or inactivation of TRP channels^51^. The inactivation kinetics seen with the R437Q and R437/K442Q mutant is not very different from the wild-type (Fig. 5C). Thus, we guess that inactivation kinetics is more directly controlled by feedback of intracellular Ca^2+^ or direct modulation via Ca^2+^ bound calmodulin to this channel but not via PIP_2_ interaction with the pre-S1domain/shoulder.

Another important issue is the binding mechanism of PIP_2_ and DAG in TRPC3/6/7 channels. These lipids essentially share the same acyl-chain structure, though neither the proximity nor the cross-reactivity of PIP_2_ and DAG binding to these TRPC channel has been determined. Our study suggests that PIP_2_ and DAG bind at independent sites, which is consistent with the dose–response curves obtained with the pre-S1 mutants in the present study. In fact, a unique DAG recognition site has recently been identified in the pore domain of TRPC3 channel^52^. These observations indicate that DAG-activated TRPC3/C6/C7 channels are accessible to both PIP_2_ and DAG within a single channel complex.

We also found that K771Q mutation completely reverses the inhibitory effect of PIP_2_ depletion without significantly affecting PIP_2_ binding, as indicated by the absence of a significant change in the value of decay (*t*_1/2_) and recovery (*τ*) from the VSP experiments (Fig. 7C). This observation implies the existence of a polarity switch for the PIP_2_ binding effect. Similar to the K771Q mutation, the R758Q mutation and K781Q/K782Q double mutation also altered the effect of PIP_2_ depletion (Fig. 2, bottom). Using Swiss-model (https://swissmodel.expasy.org), we have created a model of human TRPC6 based on TRPC4 (PDBID: 7B1G), in which residues equivalent to K771, K781 and K782 are resolved. According to this modeled structure, the three residues are located very close to the pre-S1 PIP_2_ interacting residues, most likely forming a part of the binding site (Supplemental Fig. 2). However, for K771, because this residue is positioned at a flexible outer skirt region, it is not likely to be a sole determinant of PIP_2_ binding or switching polarity yet. These findings shed light on functionality of PIP_2_, apparently localized between R758 and K782 in TRPC6/7.

This switching mechanism for PIP_2_ may be physiological relevant in vascular native cells, where the depletion of PIP_2_ effect enhances TRPC6-like currents^3,53^. As previously revealed through R721A mutation of TRPV1, a basic residue in the distal TRP box modifies their selectivity from PIP_2_ to PIP for maintaining capsaicin-activated currents^27^. This suggests that a similar effector mechanism underlies the actions of PIP_s_ in both TRPV1 and TRPC6/7 channels. Understanding how the pre-S1 domain/shoulder and the distal TRP domain orchestrate PIP_2_ regulation is an intriguing mechanism for future study.

Our present findings summarized above provide new insights into the molecular interactions between PIP_2_ and TRPC6 channel. Specifically, the pre-S1 domain/shoulder is a critical segment for PIP_2_ interaction that probably acts in concert with the TRP box and linker segment between the ARD and pre-S1. The results also reveal the switching of the mode of PIP_2_ binding action that can be made by single point mutation in the basic amino acid residues in the distal TRP box. These provide inevitable structural and functional insights into the biochemical and physiological mechanisms governing TRP channel activity.

## Materials and methods

### Plasmids and cells

A pcDNA3 expression vector encoding human TRPC6 (accession number: NM_004621) was provided by Dr. Thomas Hofmann (Institut für Pharmakologie und Toxikologie) and was transferred into a kanamycin resistant pIRES2 vector (excluded eGFP region). cDNA encoding hTRPC7 was cloned from the Human Brain Library (Invitrogen) and inserted into the pIRES2 vector. Single amino acid mutations in TRPC6 and TRPC7 were generated using a QuikChange Site-Directed Mutagenesis kit (Stratagene) according to the manufacturer’s instructions. Danio rerio VSP (DrVSP) and its inactive mutant in pIRES-eGFP vector (Invitrogen) were identical to those described previously^24,25^. To detect PIP_2_, we used PIP_2_ sensor molecules consisting of monomeric superenhanced CFP or YFP fused with a PH domain (CFPmse-PHd and YFPmse-PHd, respectively)^15^. Muscarinic type-I (M_1_R) receptor was provided by Dr. Tatsuya Haga. All PCR products were sequenced entirely.

HEK293 cells (ATCC) were maintained in Dulbecco’s modified Eagle’s medium (DMEM; Invitrogen) supplemented with 10% FBS (Gibco) and antibiotics (penicillin and streptomycin; Gibco) at 37 °C (5% CO_2_). For transfection, the cells were seeded onto poly-L-lysine-coated glass coverslips (Matsunami) in 35-mm culture dishes and transfected with a mixture of plasmid vector-incorporated DNAs using SuperFect transfection reagent (Qiagen). To screen for PIP_2_ binding sites, plasmids encoding TRPC6 and DrVSP were mixed at a 2:1 molar ratio.

### Electrophysiology

The pipette solution for whole-cell recording contained (mM): 120 CsOH, 120 aspartate, 20 CsCl, 2 MgCl_2_, 5 EGTA, 1.5 CaCl_2_, 10 HEPES, 2 ATP-Na_2_, 0.1 GTP, 10 glucose (pH 7.2, adjusted with Tris base; 285–290 mOsm, adjusted with glucose). For measurement of Ca^2+^-dependent inactivation of mutant channels in Fig. 5C, 5 EGTA and 1.5 CaCl_2_ in the pipette solution were replaced to 1 EGTA and 0.3 CaCl_2_ to reduce Ca^2+^ buffering capacity. The standard external solution contained (mM): 140 NaCl, 5 KCl, 1 CaCl_2_, 1.2 MgCl_2_, 10 HEPES, 10 glucose (pH 7.4, adjusted with Tris base; 300 mOsm, adjusted with glucose). 100 μM DIDS (4,4-diisothiocyanostilbene-2,2-disulphonic acid, 2Na, purchased from Calbiochem) was applied in the external solutions when needed. Stock solutions for 1-o-oleoyl-2-acetyl-sn-glycerol (OAG, Cayman chemical) and RHC80267 (Calbiochem) were dissolved in dimethyl sulfoxide (DMSO, Wako) at concentrations more than 1000-fold higher than used in the experiments. Carbachol (Sigma-Aldrich) was diluted in the standard external solution from its stock (100 mM) in H_2_O. The cells were continuously gravity perfused with the external solution at a flow rate of 1 mL/min. Perfusion was turned on and off using electromagnetic solenoid microvalves (Takasago Electric). The currents were recorded at a holding potential of − 50 mV. After applying 50 μM OAG in bath solution, TRPC6 currents were gradually increased and reached the peak amplitudes around 30–120 s. During this recording, strong depolarizing pulse (+ 100 mV, 700 ms) was applied every 15–25 s. For measurements of effect of PIP_2_ depletion and replenishment, several pulses (2–4) near the peak amplitudes were averaged as for the data of the individual cells.

### FRET detection

Fluorescence from voltage-clamped cells was detected using a Nikon TE300 Eclipse microscope (60×, 0.9 N.A. objective) equipped with a beam-splitter (Dual-View2, Photometrics) and an EMCCD camera (Evolve512, Photometrics). Excitation light filtered at 427/10 nm and 504/12 nm was alternately introduced via an optical fiber from a lamp-house equipped with a high-speed excitation wavelength selector (75 W xenon lamp, OSP-EXA; Olympus). Optical filters were obtained from Semrock except for the splitter (Chroma). The duration of camera exposures was 100 ms occurring within 150-ms periods of illumination at each excitation wavelength. Images were captured with an EM gain of 300 and then digitized as 512 × 512 pixels in 16-bit arrays by the microscope software (Micro-manager v.1.4). Averaged intensities from the whole-cell region (typically 20 × 20 to 40 × 40 square pixels) were analyzed to calculate FRET using a custom-written MATLAB program. Finally, the FRET ratio (*FR*) was calculated as described previously^54^.

### Docking simulation

The TRPC6 (PDBID: 6UZ8)-PIP_2_ docking process was simulated using AutoDock 4.2^55^. A 15 × 15 × 15 Å (x, y, and z) grid box was centered on the binding pocket with 0.375 nm spacing for each dimension. AutoGrid 4.2 was used to prepare grid maps. Docking parameters were set as follows: exhaustiveness = 8, num_modes = 100, and energy_range = 4. Other parameters were set at their default value.

### Confocal microscopy

HEK293 cells were transfected with wild-type TRPC6 (TRPC6_WT_) fused with CFP on the N-terminal side (CFP-TRPC6_WT_) and YFP-PHd or CFP-TRPC6_mutant_/YFP-PHd. After 48 h, the cells were seeded into a glass-bottomed culture dish (MatTek) and incubated for more than 3 h prior to imaging. Fluorescence and optical images were obtained using an inverted confocal microscope (LSM830, Carl Zeiss) equipped with a 63× oil objective lens (1.25 N.A.) at 1024 × 1024 resolution with a pixel dwell time of 6.4–25.4 µs. During confocal microscopy, the cells were bathed in the standard external solution identical to that used for the electrophysiological experiments.

### Barium influx for dose–response to OAG

Ba^2+^ influx was measured as described in previous report^56^. Briefly, transfected HEK293 cells were seeded onto glass coverslips and allowed to attach for 3 h. The coverslips were then incubated with 1 μM Fura-2/AM for 30 min and transferred to a custom-made glass bottom chamber apparatus on an inverted microscope stage (IX-83, Olympus). The solution flow rate was set to 1 ml/min using gravity-fed system. The bath solution had an identical composition used in the electrophysiological experiments only by the replacement of external calcium with barium. Cells exhibiting YFP fluorescence were selected for measurement. Fluorescence images of the cells were captured with a CCD camera (EXi Blue, QImaging) and recorded with a software (cellSens, Olympus). The 340/380 nm Fura-2 ratios from images were obtained on a pixel-by-pixel basis every 5 s for a total of 400 s. OAG (0.01–100 μM) was applied at 120 s of after the recording up to 420 s. Ratiometric *F*_340_/*F*_380_ values during the imaging were calculated by averaging the ROI intensity and analyzed by a custom written program in MATLAB (Mathworks). Delta Fura-2 ratio (Δ*F*_340_/*F*_380_) was calculated from the ratio of the maximal response minus the basal level.

### Data analysis

Electrophysiological data were analyzed using MATLAB and Excel (Microsoft). The recovery (*τ*) of the TRPC6 inward current (*I*) was determined with least-squares fits of the exponential as follows: *I* = *I*_0_ + *A*·*exp*(− *t*/*τ*), where *I*_0_ is the current size after repolarization, *A* is a scale factor, and *τ* (recovery) is the reactivation kinetics of the PIP_2_ binding. The value of *t*_1/2_ of the TRPC6 outward current decay was determined by fitting with a logarithmic equation as follows: *I* = *I*_min_ + *I*_d_/(1 + *exp*[(*t* − *t*_1/2_)/*f*_s_]), where *I*_min_ is the minimum current induced by the inhibition, *I*_d_ is the inhibited current amplitude, *f*_s_ is a slope factor, and *t*_1/2_ is a measure of the deactivation kinetics of the PIP_2_ dissociation. For confocal imaging, data were analyzed using the colocalization function in Fiji (National Institutes of Health).

Data are presented as means ± SEM. Statistical significance were evaluated using analysis of variance (ANOVA) with Tukey’s and Dunnett’s post-hoc tests for single and multiple comparisons, respectively.

## Supplementary Information

Supplementary Information.

## Data Availability

The datasets generated during and/or analysed during the current study are available from the corresponding author on reasonable request.

## References

[1] Hilgemann DW (2007). Local PIP(2) signals: When, where, and how?. Pflugers Arch..

[2] Dickson EJ, Hille B (2019). Understanding phosphoinositides: Rare, dynamic, and essential membrane phospholipids. Biochem. J..

[3] Harraz OF, Hill-Eubanks D, Nelson MT (2020). PIP_2_: A critical regulator of vascular ion channels hiding in plain sight. Proc. Natl. Acad. Sci. U.S.A..

[4] Clapham DE (2003). TRP channels as cellular sensors. Nature.

[5] Voets T, Nilius B (2007). Modulation of TRPs by PIPs. J. Physiol..

[6] Hardie RC (2014). Photosensitive TRPs. Handb. Exp. Pharmacol..

[7] Gutorov R (2022). The role of membrane lipids in light-activation of drosophila TRP channels. Biomolecules.

[8] Imai Y, Itsuki K, Okamura Y, Inoue R, Mori MX (2012). A self-limiting regulation of vasoconstrictor-activated TRPC3/C6/C7 channels coupled to PI(4,5)P(2)-diacylglycerol signalling. J. Physiol..

[9] Zhang X, Trebak M (2016). Transient receptor potential canonical 7: A diacylglycerol-activated non-selective cation channel. Handb. Exp. Pharmacol..

[10] Ko J, Myeong J, Shin YC, So I (2019). Differential PI(4,5)P_2_ sensitivities of TRPC4, C5 homomeric and TRPC1/4, C1/5 heteromeric channels. Sci. Rep..

[11] Pablo JL, Greka A (2020). Charting a TRP to novel therapeutic destinations for kidney diseases. Trends Pharmacol. Sci..

[12] Ningoo M, Plant LD, Greka A, Logothetis DE (2021). PIP_2_ regulation of TRPC5 channel activation and desensitization. J. Biol. Chem..

[13] Hofmann T (1999). Direct activation of human TRPC6 and TRPC3 channels by diacylglycerol. Nature.

[14] Ambudkar IS, Ong HL (2007). Organization and function of TRPC channelosomes. Pflugers Arch..

[15] Itsuki K (2014). PLC-mediated PI(4,5)P_2_ hydrolysis regulates activation and inactivation of TRPC6/7 channels. J. Gen. Physiol..

[16] Malczyk M (2017). The role of transient receptor potential channel 6 channels in the pulmonary vasculature. Front. Immunol..

[17] Numaga-Tomita T, Nishida M (2020). TRPC channels in cardiac plasticity. Cells.

[18] Dryer SE, Reiser J (2010). TRPC6 channels and their binding partners in podocytes: Role in glomerular filtration and pathophysiology. Am. J. Physiol. Ren. Physiol..

[19] McLaughlin S, Murray D (2005). Plasma membrane phosphoinositide organization by protein electrostatics. Nature.

[20] van Rossum DB (2005). Phospholipase Cgamma1 controls surface expression of TRPC3 through an intermolecular PH domain. Nature.

[21] Kwon Y, Hofmann T, Montell C (2007). Integration of phosphoinositide- and calmodulin-mediated regulation of TRPC6. Mol. Cell.

[22] Rohacs T, Lopes CM, Michailidis I, Logothetis DE (2005). PI(4,5)P_2_ regulates the activation and desensitization of TRPM8 channels through the TRP domain. Nat. Neurosci..

[23] Takahashi N (2014). TRPV4 channel activity is modulated by direct interaction of the ankyrin domain to PI(4,5)P(2). Nat. Commun..

[24] Okamura Y, Kawanabe A, Kawai T (2018). Voltage-sensing phosphatases: Biophysics, physiology, and molecular engineering. Physiol. Rev..

[25] Rjasanow A, Leitner MG, Thallmair V, Halaszovich CR, Oliver D (2015). Ion channel regulation by phosphoinositides analyzed with VSPs-PI(4,5)P_2_ affinity, phosphoinositide selectivity, and PI(4,5)P_2_ pool accessibility. Front. Pharmacol..

[26] Choveau FS, De la Rosa V, Bierbower SM, Hernandez CC, Shapiro MS (2018). Phosphatidylinositol 4,5-bisphosphate (PIP_2_) regulates KCNQ3 K(+) channels by interacting with four cytoplasmic channel domains. J. Biol. Chem..

[27] Ufret-Vincenty CA (2015). Mechanism for phosphoinositide selectivity and activation of TRPV1 ion channels. J. Gen. Physiol..

[28] Nilius B, Mahieu F, Karashima Y, Voets T (2007). Regulation of TRP channels: A voltage-lipid connection. Biochem. Soc. Trans..

[29] Kalia J, Swartz KJ (2013). Exploring structure–function relationships between TRP and Kv channels. Sci. Rep..

[30] Numata T (2017). Integrative approach with electrophysiological and theoretical methods reveals a new role of S4 positively charged residues in PKD2L1 channel voltage-sensing. Sci. Rep..

[31] Boulay G (1999). Modulation of Ca(2+) entry by polypeptides of the inositol 1,4, 5-trisphosphate receptor (IP_3_R) that bind transient receptor potential (TRP): Evidence for roles of TRP and IP_3_R in store depletion-activated Ca(2+) entry. Proc. Natl. Acad. Sci. U.S.A..

[32] Polat OK (2019). Contribution of coiled-coil assembly to Ca(2+)/calmodulin-dependent inactivation of TRPC6 channel and its impacts on FSGS-associated phenotypes. J. Am. Soc. Nephrol..

[33] Bai Y (2020). Structural basis for pharmacological modulation of the TRPC6 channel. Elife.

[34] Tang Q (2018). Structure of the receptor-activated human TRPC6 and TRPC3 ion channels. Cell Res..

[35] Grynkiewicz G, Poenie M, Tsien RY (1985). A new generation of Ca^2+^ indicators with greatly improved fluorescence properties. J. Biol. Chem..

[36] Chuang HH (2001). Bradykinin and nerve growth factor release the capsaicin receptor from PtdIns(4,5)P_2_-mediated inhibition. Nature.

[37] Prescott ED, Julius D (2003). A modular PIP_2_ binding site as a determinant of capsaicin receptor sensitivity. Science.

[38] Kurokawa T (2012). 3' Phosphatase activity toward phosphatidylinositol 3,4-bisphosphate [PI(3,4)P_2_] by voltage-sensing phosphatase (VSP). Proc. Natl. Acad. Sci. U.S.A..

[39] Grimm SS, Isacoff EY (2016). Allosteric substrate switching in a voltage sensing lipid phosphatase. Nat. Chem. Biol..

[40] Varnai P, Thyagarajan B, Rohacs T, Balla T (2006). Rapidly inducible changes in phosphatidylinositol 4,5-bisphosphate levels influence multiple regulatory functions of the lipid in intact living cells. J. Cell Biol..

[41] Rohacs T (2013). Regulation of transient receptor potential channels by the phospholipase C pathway. Adv. Biol. Regul..

[42] Rohacs T (2013). Phosphoinositide regulation of TRP channels. Handb. Exp. Pharmacol..

[43] Kohout SC (2010). Electrochemical coupling in the voltage-dependent phosphatase Ci-VSP. Nat. Chem. Biol..

[44] Paulsen CE, Armache JP, Gao Y, Cheng Y, Julius D (2015). Structure of the TRPA1 ion channel suggests regulatory mechanisms. Nature.

[45] Guo J (2017). Structures of the calcium-activated, non-selective cation channel TRPM4. Nature.

[46] Fluck EC, Yazici AT, Rohacs T, Moiseenkova-Bell VY (2022). Structural basis of TRPV5 regulation by physiological and pathophysiological modulators. Cell Rep..

[47] Gan N (2022). Structural mechanism of allosteric activation of TRPML1 by PI(3,5)P_2_ and rapamycin. Proc. Natl. Acad. Sci. U.S.A..

[48] Zheng W (2018). Direct binding between Pre-S1 and TRP-like domains in TRPP channels mediates gating and functional regulation by PIP_2_. Cell Rep..

[49] Yin Y (2019). Structural basis of cooling agent and lipid sensing by the cold-activated TRPM8 channel. Science.

[50] Moon CP, Fleming KG (2011). Side-chain hydrophobicity scale derived from transmembrane protein folding into lipid bilayers. Proc. Natl. Acad. Sci. U.S.A..

[51] Mercado J, Gordon-Shaag A, Zagotta WN, Gordon SE (2010). Ca^2+^-dependent desensitization of TRPV2 channels is mediated by hydrolysis of phosphatidylinositol 4,5-bisphosphate. J. Neurosci..

[52] Lichtenegger M (2018). An optically controlled probe identifies lipid-gating fenestrations within the TRPC3 channel. Nat. Chem. Biol..

[53] Large WA, Saleh SN, Albert AP (2009). Role of phosphoinositol 4,5-bisphosphate and diacylglycerol in regulating native TRPC channel proteins in vascular smooth muscle. Cell Calcium.

[54] Butz ES (2016). Quantifying macromolecular interactions in living cells using FRET two-hybrid assays. Nat. Protoc..

[55] Morris GM (2009). AutoDock4 and AutoDockTools4: Automated docking with selective receptor flexibility. J. Comput. Chem..

[56] Condrescu M, Chernaya G, Kalaria V, Reeves JP (1997). Barium influx mediated by the cardiac sodium–calcium exchanger in transfected Chinese hamster ovary cells. J. Gen. Physiol..

