# Supplementary material for: Critical contributions of pre-S1 shoulder and distal TRP box in DAG-activated TRPC6 channel by PIP2 regulation
Source: Sci Rep. 2022 Jun 24;12:10766. doi: 10.1038/s41598-022-14766-x (PMC9232555; doi:10.1038/s41598-022-14766-x)
Supplement: Supplementary file 1 — Supplementary Information. [file 41598_2022_14766_MOESM1_ESM.pdf]

Supplementary information for "Critical Contributions of Pre-S1 Shoulder and Distal TRP Box in DAG-activated TRPC6 Channel by PIP_2 Regulations"

Masayuki X. Mori[1,3†], Ryo Okada[1,2], Reiko Sakaguchi[1], Hideharu Hase[3], Yuko Imai[4], Onur K. Polat[3], Satoru G. Itoh[5,6], Hisashi Okumura[5,6], Yasuo Mori[3], Yasushi Okamura[7], Ryuji Inoue[4]

[1] Laboratory of Bio-materials and Chemistry, School of Medicine, University of Occupational and Environmental Health, Japan

[2] Human Information and Life Sciences, School of Health Sciences, University of Occupational and Environmental Health, Japan

[3] Laboratory of Molecular Biology, Department of Synthetic Chemistry and Biological Chemistry, Graduate School of Engineering, Kyoto University, Japan

[4] Department of Physiology, School of Medicine, Fukuoka University, Japan

[5] Exploratory Research Center on Life and Living Systems / Institute for Molecular Science, National Institutes of Natural Sciences, Japan

[6] Department of Structural Molecular Science, SOKENDAI (The Graduate University for Advanced Studies), Japan

[7] Laboratory of Integrative Physiology, Department of Physiology, Graduate School of Medicine, Osaka University, Japan

[†]Corresponding author

Correspondence should be addressed to M.X.M (e-mail: mxmori@med.uoeh-u.ac.jp)

Contact address

Masayuki X. Mori Ph.D. Professor

Laboratory of Bio-materials and Chemistry, School of Medicine, University of Occupational and Environmental Health

1-1, Iseigaoka, Yahatanishi-ku Kitakyushu-shi, Fukuoka, 807-8555, Japan

Phone: +81-93-603-1611, Fax: +81-93-693-992, E-mail: mxmori@med.uoeh-u.ac.jp

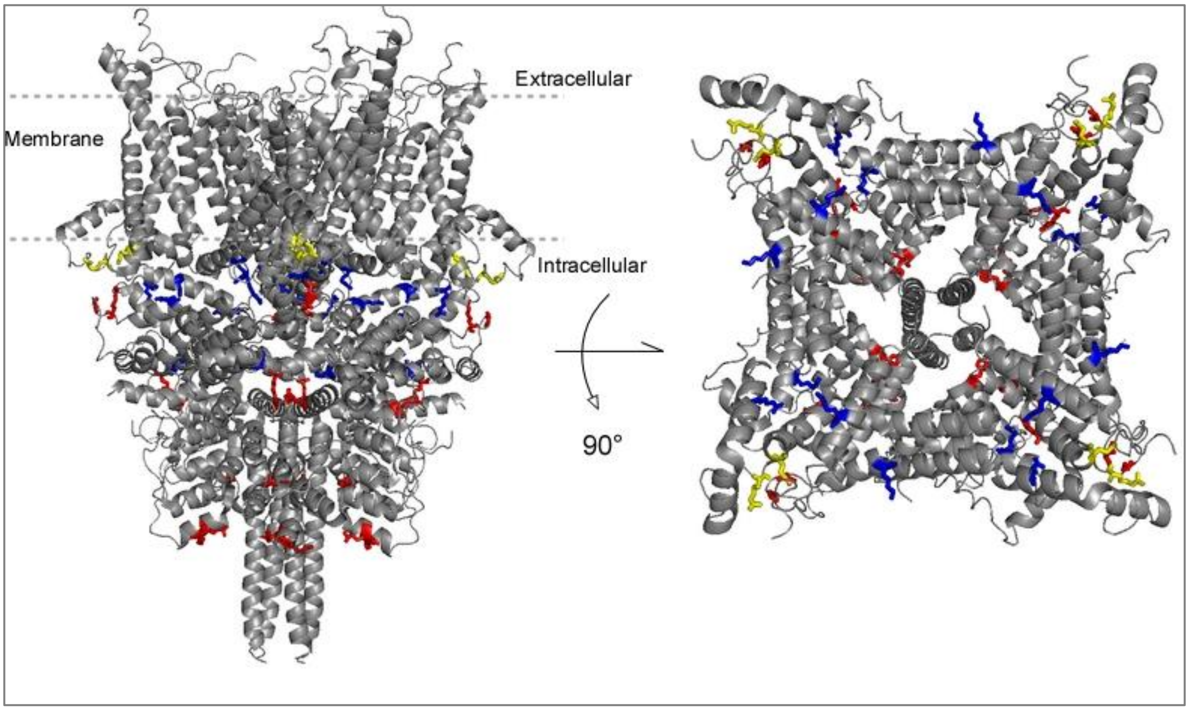

Supplemental figure 1. A map of deactivation and reactivation changes in hTRPC6 gating upon PIP_2 deletion or replenishment.

Side view (left) and top view (right). In the top view, transmembrane region from 443 to 727 a.a. is omitted for visualization. Mutations that cause faster decay ($t_{1/2}$) and slower recovery (τ) compared with wild-type TRPC6 channel (Bai *et al*., 2020, *elife*) are depicted in blue and red color sticks, respectively. R437 and K442 residues which are altered both kinetics are highlighted in yellow sticks. Blue and yellow colored residues are located in the inner leaflet of the transmembrane domain and in the four corners of the TRPC6 channels (PDB: 6UZ8). Contrary, red residues are scattered in the cytoplasmic domain. The distal TRP box is unsolved in this structure.

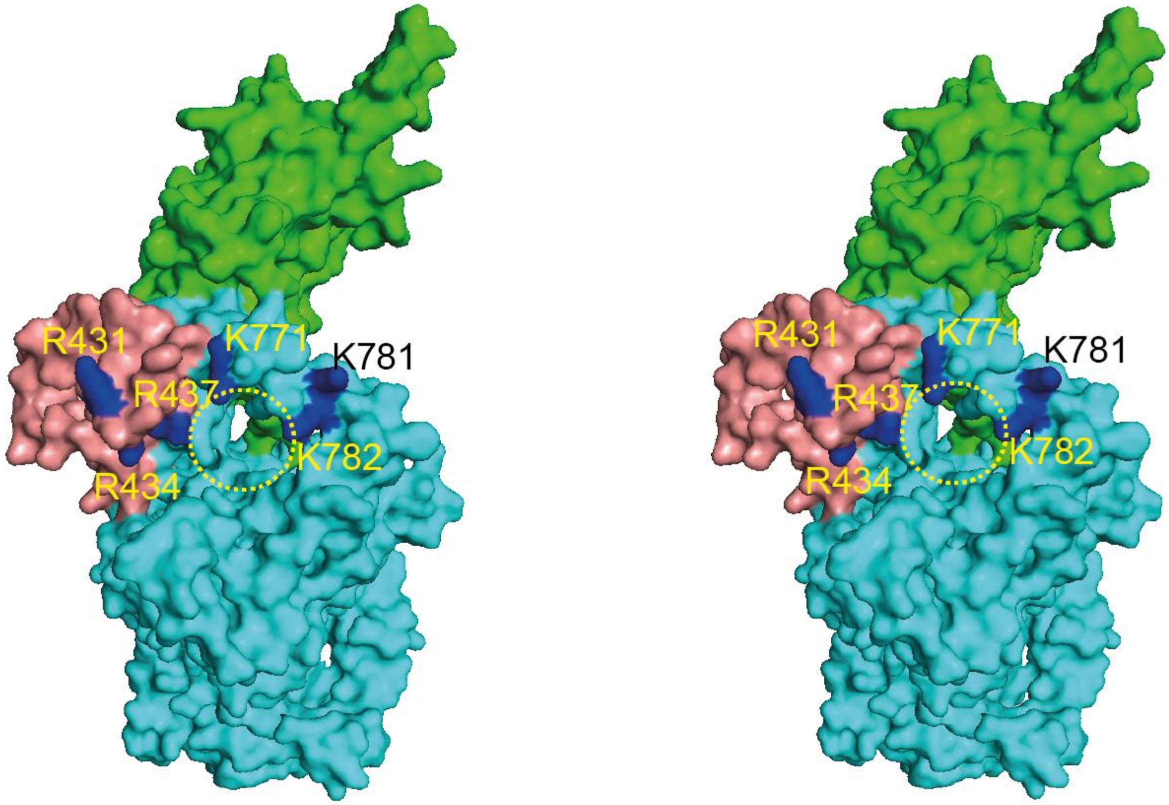

Supplemental figure 2. Stero-side view of modeled pre-S1 structure of TRPC6 with distal TRPbox.

Surface representation of the modeled structure of hTRPC6 based on TRPC4 (PDBID: 7B1G, Vinayagam *et al.*, 2020, *elife*) using Swiss Model (https://swissmodel.expasy.org). Residues K771, K781 and K782, which were not resolved in the original structure, are presented in dark blue along with K431, R434, and R437. The dotted yellow line indicates the putative PIP_2 binding cavity. Red, green, and light blue areas are pre-S1 domain, transmembrane segment, and the distal TRP domain, respectively (supplemantal material, file name: C6modeledonC4.pdb (for pymol (https://www.pymol.org) .pse)).

References

Bai, Y. *et al*, Structural basis for pharmacological modulation of the TRPC6 channel. *elife* 9, e53311 (2020).

Vinayagam,D. *et al*, Structural basis of TRPC4 regulation by calmodulin and pharmacological agents. *elife* 9, e60603 (2020).
